# Wound Bacteriology1.Abstract from an article written in Aug. 1918, to be published by the American Red Cross for the Medical Corps of the United States Army. In this abstract most of the technical details are omitted.

**Published:** 1919-01

**Authors:** Theodore C. Beebe


### WOUND BACTERIOLOGY[1]

### AT

### EVACUATION HOSPITAL No 1, AMERICAN E. F., FRANCE

By Captain THEODORE C. BEEBE, M. C.

The study of the bacteriology of war-wounds at an advanced hospital, as compared with that at a base hospital, requires the consideration of several factors.

In the first place there is the question of the period of time that elapses between the hour when the patient is wounded, and the hour of his arrival in the hands of the surgeon who is to treat him. Secondly; recognition of the fact that, under normal conditions, when a patient is transferred to a base hospital he has already been operated upon, and the bacterial flora may be quite different from that present in the original wound. Thirdly; in times of great activity the character of the bacteriologic study of wounds at an advanced hospital must be modified to suit existing conditions. At the same time a number of cases, previously untreated, may be sent directly through to a base hospital, which will be temporarily assuming the functions of an advanced hospital, so far as the treatment of the patient is concerned.

We will first consider the relations which should exist between the surgical services and the wound bacteriologist. It is probably true that never before in the history of surgery have the surgeon and the bacteriologist been brought into such intimate daily contact. The surgeon wishes to know not only the character of the infection, but the possibility and the time for performing a primary, delayed primary, or a secondary closure. It has been demonstrated beyond a doubt that the surgeon who closes a wound, apart from primary closures, without all the available bacteriological data at hand, may assume an unjustifiable responsibility. On the other hand, when the surgeon neglects to close a wound, which from the smears and cultures might be done, he may deprive the patient of an opportunity for a quick recovery, and add a great expense to the government in the care and treatment of the patient. In addition to which, it must be remembered that the functional result of a wound healing by first intention after a primary, or delayed primary closure is at almost all times better than that following secondary closure, or healing by granulation.

1. Abstract from an article written in Aug. 1918, to be published by the American Red Cross for the Medical Corps of the United States Army. In this abstract most of the technical details are omitted.

For an intelligent understanding between the two services, it is very necessary to have complete co-operation. For this reason I believe that it is essential for the bacteriologist to have a certain amount of clinical experience in the appearance and progress of war wounds, and, on the other hand, for the surgeon to have frequent consultations with the bacteriologist.

At this hospital the bacteriologists are assigned to the different team surgeons, make ward visits with them, observe the progress of the wounds, take such cultures and specimens, under the direction of the surgeon, as are necessary, and, in general, learn as much about the cases as possible. By following this method they develop a more intelligent interest in their routine work than they would otherwise have. The surgeons are accustomed to come to the laboratory to see cultures, and to discuss the conditions of the wounds, and the possibility of their closure.

It should be understood, of course, that this applies only to normal times. When there is unusual activity both services have enough to do in taking care of their necessary work.

Before taking up the discussion as to the procedures which are most applicable to the bacteriologic study of war wounds, it may be inquired what are the most important periods of a wound when cultures and smears should be taken. Should the culture be taken from the wound on entrance, before operation, during operation, after operation, or at the first subsequent dressing? Here, as in many other questions of routine, it depends upon how much time the bacteriologist has at his disposal. If he has not too much to do he may take cultures and smears at all these times, and thereby get a very accurate impression of the bacterial condition of the wound.

It has been laid down as a maxim by many of the French observers that it is impossible to find bacteria microscopically in a bleeding wound less than six or eight hours old. Up to that time the wound is practically sterile, the few bacteria taken in by the projectile not having had time to proliferate. Cultures taken during this interval may also prove to be sterile, but we have several times obtained growths from wounds four hours old.

We have taken cultures from a series of wounds before, during and after operation, and have found them to be of great comparative interest, but not necessarily of corresponding value. In many cases during operation, we have taken direct cultures from the tissues and exudates, as well as projectiles, including stones, fragments of bone, and pieces of clothing, all of which were cultured in suitable media. Sections of devitalized muscle, or other tissue, removed during operation have also been carefully examined. What then is the best time, from the point of view of the surgeon, for taking cultures and smears, and why? It is very obvious that the bacterial flora of a wound before, and even during an operation, will be entirely different from that found later, owing to the very nature of the operation. The modern operation of debridement and excision presupposes the removal of a comparatively wide margin of healthy tissue containing all the devitalized and infected tissues, extreme care being used to prevent the contamination of the walls and margins of the cavity thus produced. In this mass of tissue thus removed, it is assumed that all of the infecting organisms are included. Assuming that the operation has been thoroughly carried out, it has been demonstrated in a large number of cases that the bacteria have been entirely removed, or so few left behind that they have been prevented by the organism from proliferating. The determining of the original bacterial flora in such a case is of much greater interest to the bacteriologist than it is to the surgeon, for the latter may take it for granted that he no longer has the original flora to deal with. Should a culture be taken, then, at the completion of the operation, whether the wound is to be closed or not? Our position is that it is of no use at this time, because if the operation has been thoroughly done there are so few bacteria left behind that it is extremely unlikely that any growths will be obtained. In such a case the surgeon will perform a primary closure, or not, depending upon the anatomical difficulties, or such other factors as he may decide. When the bacteriologist is present at such an operation he may take direct cultures from the infected tissues in situ, or after their removal, as well as from foreign bodies, for the purpose of determining the original infection. Here, such knowledge is of more value to him than it is to the surgeon.

We now come to what I consider to be the most favorable time for taking cultures and smears for bacterial counts. In this view I am supported by a large part, if not most, of the French bacteriologists and surgeons.

I believe that, if the surgeon decides to perform a primary closure, the ideal method is to place at the most dependent part of the wound a few strands of silkworm gut or horse-hair, leading to the deepest part of the wound. At the first dressing, which should be done within twenty-four hours, these should be removed by the surgeon, the bacteriologist being present if possible. There follows their removal the escape of a certain amount of serum which will most certainly be infected if bacteria have been left behind after operation. From this serum the bacteriologist will be able to secure his cultures and smears. A stained smear will show the presence or absence of bacteria, and in the presence of such evidence the surgeon can very shortly decide whether the wound should be immediately reopened or not. On the other hand he may decide, on the clinical condition of the wound, to await the result of the cultures before reopening. It has been objected by some surgeons that it is unwise to introduce a foreign element into an otherwise aseptic and firmly-closed wound, with the formation of a sinus, however small, resulting from the removal of the strands. It is my firm belief that the chances of infecting secondarily such a wound are much overbalanced by the element of safety thus obtained. In the hands of surgeons who have had wide experience the progress of such wounds may be at times determined by the clinical picture, but I have known of several cases which have had to be reopened, with streptococcal infection, under such conditions. In consideration of the varying ability and amount of experience of our surgeons, I believe that the procedure outlined above will be a safer routine to follow, than to depend on clinical evidence alone.

I have said above that I consider this to be the ideal method. The fact remains, however, that at this hospital this method has not been a routine procedure. It has been followed in a certain number of cases with very good results. There have been a large number of cases of primary closure, on the other hand, which have been entirely successful without any bacteriological control. The surgeons are, however, men of unusual experience in the treatment of war wounds, and this must be borne in mind in deciding on the merits of the two methods. It may be mentioned here that it is a definite rule that no primary or delayed primary closures are to be immediately evacuated from the hospital.

If for one reason or another the wound is not closed at the original operation, a culture and smear taken at the first dressing will give the surgeon such information as will allow him to perform a delayed primary closure, or not, as he may decide.

It is of course evident that the preceding remarks apply only to cases of very recent injury, where infection has not had sufficient time to become well established, and a large number of cases are of such a nature. There are, however, a large number of cases where the time that elapses between injury and the patient's arrival in the hands of the surgeon is much longer. Here we have infection well established on arrival. There are also many cases where the infection is of a rapidly spreading, or fulminating type. In both instances it is very doubtful whether the surgeon would consider himself justified in performing a complete operation of debridement and excision. He would then clean up the wound as much as possible and establish some form of irrigation, such as Carrel's method. Here then, the original infection of the wound persists, and it is of the utmost importance for him to know what the infection is, both aerobic and anaerobic. In such a case cultures are taken from the most likely-looking foci of infection.

### Procedure for the Bacteriologic Examination of War Wounds.

For such an examination one should employ all the aids which may lead to a correct diagnosis of the bacteriologic condition. These are, not in order of importance, the hanging-drop, the stained smear on a glass slide, aerobic and anaerobic cultures, and the leucocytic index.

The hanging-drop reveals the presence or absence in the original exudate, or a later liquid culture medium, of cocci, in chains or not, and of bacilli. The presence or absence of motility and of spores in the latter should be noted. The technic and significance will be discussed later.

The stained smear on a glass slide for the bacterial count, not only for the relative number, but for the relative proportion, between cocci and bacilli, is of great importance.

### The Hanging-Drop.

The information secured from the hanging-drop from the original, or later, exudates, or from a fluid culture medium, may be of great importance. If taken for the academic value of determining the original infection, or as a guide to the advisability of a primary closure, the information will depend largely on the length of time that has elapsed since the wound was received. In the first ten hours, as has been said, there is usually very little proliferation of bacteria, but there may be enough to be of definite assistance. Beyond this time growth is usually well assured.

### The Stained Smear.

The value of the stained smear for the determination of the bacterial count, and the bacterial index, has been the subject of much controversy. I believe that it has a great deal of value when the results are correctly interpreted. By this I mean that the results must be taken as a relative index, and not as a definite numerical entity. The objection has been frequently raised, and properly so, that it is impossible always to obtain smears of the same uniformity and thus the readings will constantly vary. As a matter of fact, this objection has been shown to be more or less theoretical. A series of smears of varying density from the same specimen show a striking similarity in the count, especially when the number of bacteria present is very low, or very high, per field. It is quite true that in the intermediate ranges the counts will vary to a greater or less degree. Fortunately, we are more concerned about the low counts, as here it is a question of the advisability of closing the wounds. When the count is high, anywhere from twenty per field to infinity, we know that the wound cannot safely be closed, regardless of what the cultures may show. But a series of counts, every day, or every other day, showing a steady diminution in the number, is evidence that the infection is diminishing, and that the wound may soon be in condition to close, other factors being favorable.

It has sometimes been stated that a clean-appearing wound may be closed on the clinical appearance alone. This is not always true, as many lamentable failures have shown. It is frequently apparent that wounds infected with virulent streptococci present a very clean appearance, especially when they have been treated by Carrel's method, thus altering the natural appearance of the wounds.

It has been urged against the necessity of following wounds by bacterial counts, that when a wound appears to be ready for closure it requires only a negative culture, or series of cultures, to confirm this belief. These should be secured in any event before the closure is made, even when the bacterial counts are also made. Many times a few bacteria in a wound will give profuse growths in the cultures, and we thus have no guide as to the number in the wound unless the bacterial counts are made. On the other hand, a series of low counts, while the cultures show no hemolyzing cocci, or anaerobic bacilli, is a direct bacteriologic indication for closure.

It has also been objected that, in the course of treatment, the count of one day may at times show a great increase from that of the previous day, and thus is of no value. My observation of such cases leads me to the conclusion that usually such a sudden increase is due to a break somewhere in the Carrel technic, especially in the application of the dressings.

The process of making the smear and determining the number of bacteria per field is easily and very quickly done. It is an important aid, though not conclusive in itself, and should always be adopted as part of the routine. In addition to the count, the bacterial index, or the proportion of cocci to bacilli, is always of interest, but not necessarily important. This can be very easily determined while the count is being made.

### TECHNIC.

Sometime ago I observed in several cases, where the count should theoretically have been low, what appeared to be a count of infinity of cocci in every field. They were all of approximately the same size. Fresh stains were made up but the results were the same. On inquiry, the cases were all found to be suffering from surgical shock. At about the same time, at autopsies performed here on cases dying in extreme shock, it was demonstrated that the blood contained an enormous amount of free fat. It immediately occurred to me that we were dealing with the same condition here. The cultures proved to be negative, or nearly so, and the fat droplets disappeared as the cases improved. So far as I am aware this condition has not been described before. Whether the presence of these fat droplets in blood-smears from cases of surgical shock, with their relative enumeration, might be of any value in the study of shock, is an interesting question which might be considered. In these cases the swabs were taken from the depths of wounds, where it was fairly safe to assume that the fat was not extravasated directly from the subcutaneous tissues, nor from bone-marrow. The only fat-solvent tested on the slides was ether, and the droplets were found to be dissolved in this solvent. No other fat-stains, such as Scharlach red or Sudan III, were tried by the writer, but it is hoped that further investigation with other solvents and stains can be made in subsequent cases. Sections of lung-tissue, taken from cases dying from shock, were examined by Captain Wayne W. Bissell, Pathologist to Evacuation Hospital No. 1. These showed the presence of great amounts of fat, stained by Sudan III, in the blood vessels.

Another source of confusion occasionally encountered is the presence of minute, apparently stained droplets from another source. Part of the technic for the Carrel treatment consists in the placing of strips of gauze impregnated with sterile vaseline about the margins of the wound, to protect the surrounding skin from the action of the Dakin's solution. Some of this vaseline at times is introduced into the wound and may be picked up by the swab and thus transferred to the slide. We then find these minute stained droplets which may be mistaken either for cocci or for deposits in the stain. This possibility should be borne in mind.

The method of making a bacterial count should be, and probably is, perfectly familiar to all of our bacteriologists. At this hospital we are accustomed not only to make the counts, but also to consult with the surgeons as to the advisability of closing the wounds. We are very frequently asked if it is safe to close a wound on the evidence of the smears and cultures, other factors being favorable. Our advice is, usually, that if the count shows one bacterium in two fields, or less, for two or three successive counts, and the cultures show no hemolyzing cocci and very few anaerobic bacilli, the wound may be closed. We have occasionally advised closure where it was most important that it should be done as soon as possible when the count showed four per field. Here, however, the cultures showed one or two small colonies containing a non-pathogenic diplococcus. Fortunately these cases all healed perfectly, but such advice should only be given in cases of great necessity, and under the careful watching of the surgeon. As was stated above, no wound should be closed on the advice of the bacteriologist until the low counts have been confirmed by negative cultures, or cultures showing only a rare colony of non-hemolyzing cocci, and very few anaerobic bacilli.

(The technical details of the culture media used are then given, with a description of the various organisms commonly found.)

### Aerobes.

In the presence of cocci in war wounds we are immediately confronted with many confusing factors. We find cocci in pairs, in groups, and in chains under so many different conditions and combinations, that at first it seems impossible to make any clear differentiation. This applies not only to cocci as found in cultures, but also to cocci as seen in the smear and hanging-drop. It has been our custom to call as streptococci only such chain-forming cocci as produce hemolysis in blood (S. hemolysans). All others, with the exception of S. viridans, we designate as " cocci in chains ". It should be understood, of course, that this is purely a local nomenclature. Staphylococci, other than Staph. aureus, we speak of as " cocci in groups ".

On examination of young cultures, then, both in the liver-peptone water and on the agar slant, from the great majority of war wounds showing a positive growth, we find cocci single, in pairs, in groups, or in chains. The hemolyzing cocci we assume, in the absence of contradictory evidence, to be virulent, while the non-hemolyzing cocci are not virulent, and act in the wound simply as saprophytes. Under what species may we classify the non-hemolyzing cocci? It has been found that all the varieties of cocci mentioned in our list may at different periods of their growth and under different conditions of growth, appear singly, in pairs, in groups or in chains. Furthermore, of the two elements of a diplococcus, both may be alike, one may be round, while the other may be lance-shaped, or an elongated form simulating a bacillus. In fact, all the different combinations may be seen at times. They may also be encapsulated or not. We will consider first the three varieties which we are continually finding, and endeavor to differentiate them : streptococcus, enterococcus, and diplococcus griseus non liq. Microscopically they may be, and usually are, identical in young cultures.

Aside from streptococci and staphylococci, which may be distinguished by their hemolyzing action on blood, we see that the enterococcus, and the diplococcus griseus, may present similar morphological characteristics. Dr. Legroux of the Pasteur Institute believes that they may be forms of a virulent pneumococci. It is possible that a study of their behavior toward bile might throw more light on the subject. We have as yet made no attempt to classify the various strains of streptococci found, with the exception of the streptococcus viridans. We have found this organism three times, once in pure growth from a blood culture. The *Micrococcus candidus* appears to be a variety of the staphylococcus albus, probably the same as the staphylococcus epidermidis albus. As compared with wounds in civil practice, where the staphylococcus aureus is, perhaps, the organism most frequently found, it has been noticed here that it is comparatively seldom found in fresh wounds. Most of the staphylococci found seem to be skin cocci, so-called. Enterococci and the diplococcus griseus are facultative anaerobes, and at times grow in a thick cloud of fine lenticular colonies throughout deep agar. This is a source of great annoyance, making it very difficult to pick out colonies of the anaerobic bacilli.

The aerobic bacilli found in war wounds are many, some varieties of which appear to be rare in wounds encountered in civil practice. Legroux has stated that the aerobic bacteria most frequently seen in fresh war wounds are four in number : streptococci, staphylococci, bacillus pyocyaneus, and the bacillus of Friedlander. This has not been our experience, but it is quite possible that the flora of one region may be quite different from those of other regions. This may be accounted for by many different factors, such as the character of the soil, and the conditions and degree of cultivation.

### Pathogenicity.

Many of the anaerobic bacilli appear to be pathogenic in themselves, while others seem to be pathogenic only in symbiosis, either with other anaerobes or some of the aerobes. It has been demonstrated that Bac. welchii, the Vibrion septique, Bac. œdematiens, and the Bac. histolyticus are pathogenic in themselves, while most of the other anaerobic bacilli appear to play what may be termed preparatory roles.

The bacilli which seem to be most prominent in the causation of gas bacillus infection are the Bac. Welchii, the Vibrion septique, and the Bac. œdematiens (Bac. bellonensis?). They may be present alone in the lesions, or may be seen in various combinations with the other pathogenic or non-pathogenic anaerobic bacilli. Some of these latter appear to aid in the process by producing putrid conditions, or gas, or both, in the wounds.

The salient feature of war wounds, or battle casualties, as they are sometimes called, is the great amount of subcutaneous destruction of tissues, caused by the passage of the projectile. The external appearances of the wounds of entrance or exit afford no indication as to the amount of internal harm done. The waves of centrifugal force set in action by the passage of the projectile not only cause an unusual amount of devitalization of the neighboring tissues, but also scatter widely minute particles of clothing, hair, soil, spicules of bone, and, most important of all, bacteria. Sections of tissue taken from the track of a projectile show such particles and bacteria, scattered throughout the tissues to a depth of one, two or more centimeters. With the focus of infection in such devitalized tissue already at hand, it is readily apparent how important the operation of debridement and excision of such tissue becomes. The soil of the terrain over which the troops are fighting is, for the most part, highly fertilized, and contains large numbers of bacteria, particularly the anaerobic bacilli. The clothing and skin necessarily become contaminated with this soil, and it is for this reason that the projectile in its passage carries with it the means of infection.

We have seen that in the first six or eight hours bacteria are not usually found in any considerable number, microscopically, in smears taken from wounds. Cultures made at the same time usually show only a few colonies. The first bacteria to appear are usually cocci, either singly, or in pairs, followed by the presence of Gram positive bacilli, of which the Bac. welchii seems to be the most rapidly growing. At the end of twenty-four hours the growth of both aerobes and anaerobes is well established in the great majority of untreated wounds. From the fourth to the tenth day the anaerobic bacilli diminish in growth, and in virulence. During this time the cocci also show less growth, some of them tending to disappear, while the more virulent forms such as the hemolyzing cocci persist. During this period we frequently note the appearance of other bacteria, such as the Bac. coli, Bac. proteus, and the Bac. pyocyaneus. Also during this period we may have the development of certain anaerobic bacilli, such as the Bac. sporogenes, causing putrefactive changes in the wound.

In a wound treated, either by operation (debridement and excision), or by Carrel's method, we see quite a different picture.

After operation, if properly performed, cultures show rare colonies, usually of cocci, single or in pairs. Owing to the removal of devitalized tissues, which offer the most favorable factor for their growth, we find few, if any, anaerobic bacilli. In such an event, if the cocci are not of the hemolytic type, they usually disappear at an early date, and the case is usually ready for an early closure.

A certain number of cases are too severe for a radical debridement and excision of tissues. In such a case the surgeon removes as much of the devitalized tissues as possible, and institutes the Carrel system of drainage and irrigation. Here, we may have enough of a focus of devitalized tissue and bacteria left behind to allow of the development of a certain number of both aerobic and anaerobic bacteria. Owing to the access of oxygen and the elimination of toxins, and the products of putrefaction, we usually have a rapid diminution, both in number and virulence, of the bacteria. A certain number of cases, however, under such conditions, will show a development of anaerobic bacilli leading to gas gangrene.

Aside from the question of gas gangrene the most important complication we have to deal with is infection with hemolytic cocci. This is important in any case, but especially so when there is infection of bone tissue. It is practically an axiom that every case of sinus leading down to a bone, in a wound of long standing, is infected with hemolytic cocci, usually streptococci. In such an event we should *always* advise against closure of the wound until several repeated cultures show the absence of hemolytic cocci.

### Gas Gangrene.

The subject of gas gangrene is too extended to allow of more than a short resume in the present article.

No case of gas gangrene caused by aerobic bacteria has yet been discovered here. All cases of gas gangrene are caused by anaerobic bacilli, singly, in combination with other anaerobic bacilli, or with aerobic bacteria. The majority of cases of gas gangrene are caused by the action of two or more anaerobic bacilli. The cases seen at this hospital are caused more frequently by the Bac. welchii than by any others. The bacillus most frequently found by us in combination with the Bac. welchii is the Bac. sporogenes. In several cases the determining organism, either alone, or in combination with others, was the Vibrion septique. We have not as yet demonstrated the presence of the Bac. œdematiens in any of our cases. Among the aerobes found in cases of gas gangrene, the most frequent are non-hemolytic cocci, usually of the diplococcus griseus type. Enterococci are also frequently found. We frequently find also the Bac. proteus, member of the Bac. subtilis group, and the Bac. mesentericus. Hemolytic streptococci or staphylococci have been found in a comparatively small number of fresh wounds. The presence of anaerobic bacilli in blood cultures has not as yet been demonstrated in any of our cases.

For the clinical classification of the different forms of gas gangrene, that of Weinberg and Seguin seems to me the most logical. They classify them into the following groups :

1st The classical type.
2nd The toxic type.
3rd Mixed types.

All of these three may be complicated with the putrid varieties.

1. *The Classical Type.*

In this form of gas gangrene there are two principal infecting organisms, the Bac. welchii and the Vibrion septique. The cardinal symptoms of this form are : 1st, the production of bubbles of gas in the tissues; 2nd, sometimes, but not always, crepitation in the overlying tissues; 3rd, bronzing of the skin; 4th, occasionally the formation of bullae in the adjacent skin; 5th, loss of contractility and change of color in the muscle fibres; and, 6th, in fatal cases, septicemia. Among our cases, all the foregoing symptoms have not been present in any one case. There may be any combination of these symptoms present, depending upon the location and the severity of the wound. In addition to the above-mentioned symptoms there may be present an odor of butyric acid, or a putrid odor. When there is a large amount of gas in the tissues the infecting organism is usually the Bac. welchii. In cases where the Vibrion septique has been found to be the principal infecting organism, there is usually to be found less gas production than where the Bac. welchii is the cause. The most important single symptom is the loss of contractility, and the change in color of the muscle fibres. In the great majority of cases of this type of gas gangrene the Bac. welchii has been demonstrated to be the causal factor.

2. *The Toxic Type.*

In these cases the predominating features are general symptoms of intoxication and a progressively invading œdema of the tissues, which may mask the production of gas in the tissues. Septicemia is rare. The muscles show hyperemia and are œdematous. The intercellular tissue presents a gelatinous white œdema. There is no putrid odor. Weinberg and Séguin consider that these cases are usually caused by the Bac. œdematiens, but they cite one case where, in addition to the white œdema, there was some gas in the wound, and crepitation near the wound. Cultures showed the presence of the Bac. welchii alone. We have not as yet seen any cases of this type in this hospital.

5. *Mixed Types.*

The Bac. welchii, or the Vibrion septique, may be present in a wound in combination with the Bac. œdematiens. In such a case one might observe the existence, at the same time, of an extensive gaseous crepitation and a marked white œdema. Such cases have been reported, but we have not observed them at this hospital.

Several putrid varieties of gas gangrene have been observed. We have found one case in which the Bac. sporogenes was the only anaerobic bacillus present. It may be, and usually is, present in combination with the Bac. welchii, the Vibrion septique, or the Bac. œdematiens. It is by far the most common organism producing putrid conditions in wounds, but we have also found present the Bac. bifermentans and the Bac. putrificus contributing to similar conditions.

The question naturally arises why, when practically all wounds are infected with anaerobic bacilli, does gas gangrene occur in some untreated cases and not in others. The reason must be sought in the consideration of two main factors: mechanical and bacteriological.

It is easily understood that, in addition to the foreign bodies carried into a wound, one must take into consideration not only the traumatism of soft parts, but also that of bones, vessels, and nerves. We have seen that spicules of bone may be scattered widely throughout the neighboring tissues, and may carry with them numbers of bacteria, particularly the anaerobes. If these spicules of bone are not thoroughly removed we have present a very good focus of infection in devitalized tissue, without which anaerobic bacilli do not tend to develop. Also, a projectile fracturing a bone may carry into the medulla enough bacteria to give rise to an infection, and, in such an event, local treatment is almost powerless to prevent the development of severe infection.

Wounds involving the larger arteries and veins are extremely important, inasmuch as the local blood supply of the affected part is diminished, adding to the already devitalized condition of the soft parts, and bringing less oxygen to the tissues. Dressings and splints applied too tightly may also cause a similar condition.

Wounds involving nerves, so far as they affect the nutrition of the tissues, may also aid in the further devitalization of the tissues, favoring infection.

Consideration of these factors will partially explain why, in a certain number of cases, the development of gas gangrene is delayed in its onset.

In the consideration of the bacteriologic factors leading to the production of gas gangrene we encounter several elements. One of the most important of these is the association, or symbiosis, of bacteria, both aerobic and anaerobic. It is a debatable point as to how far the presence of aerobes in a wound tends to increase the virulence of anaerobic bacilli. Researches of various workers in this field lead to quite different conclusions. It is generally admitted, however, that the presence of aerobic bacteria tends to favor the growth of the anaerobes by depriving the affected tissues of their oxygen. It is also not improbable that the products of metabolism formed by the aerobes, and their action on the tissues, may be a favoring element. When two or more types of anaerobic bacilli are found in a wound, the action of one, such as the Bac. welchii or the Vibrion septique, may be increased by the putrefying effect of such an anaerobe as the Bac. sporogenes, or the histolyzing effect of the Bac. histolyticus.

Anaerobic bacilli do not grow readily in healthy living tissue, but in war wounds we find ideal conditions for their development. Here we have already devitalized tissue and a nidus of bacteria which will produce the conditions under which the anaerobic bacilli will grow most favorably, if the wound be left untreated. If the wound is operated upon at an early hour, and a complete debridement and excision of all the devitalized tissues and foreign bodies is made, a process of gas bacillus infection may be prevented. If it has already begun, it may be arrested by supplying oxygen, in the form of air, to the wound, and the constant elimination by irrigation, as in Carrel's method, of dead tissues, and the products of putrefaction.

I wish to express here my deep appreciation of the valuable aid given me by Dr. Weinberg of the Pasteur Institute. In addition to much instruction and information, he has supplied me with many pure cultures of anaerobic bacilli, and various agglutinating sera, without which the work which has made the present article possible could not have been accomplished. I am also indebted to Drs. Séguin and Legroux of the Pasteur Institute, and Drs. Policard, Weissenbach, and Vaucher of the French army laboratories, for much valuable information.

NOTE. — Since the above article was written, many smears from wounds in cases suffering from extreme surgical shock have been examined by the writer. The smears on glass slides have been fixed in formalin vapor for 45 minutes. Part of them have then been subjected to the solvent action of either ether or xythol for 15 minntes. All of the slides have then been stained either with Sudan III or Neutral Red. The slides untreated by a fat-solvent showed the presence of large amounts of small fat-droplets, while those treated as described above showed no red-stained fat.

